# Seizure Presentation of a Grade II Astrocytoma in a Patient With Ollier's Disease: A Case Report and Brief Review

**DOI:** 10.7759/cureus.102435

**Published:** 2026-01-27

**Authors:** Marissa Vail, Hunter Slosser, Robert Steele, Brian Curtis, Daniel Henry

**Affiliations:** 1 Osteopathic Medicine, Kansas City University of Medicine and Biosciences, Joplin, USA; 2 Emergency Department, Freeman Health System, Joplin, USA; 3 Neurological Surgery, Freeman Health System, Joplin, USA

**Keywords:** astrocytoma, enchondroma, enchondromatosis, glioma, idh1 mutation, isocitrate dehydrogenase 1 gene, ollier's disease, seizure presentation

## Abstract

Ollier's disease (enchondromatosis) is a dysplasia of cartilage characterized by multiple enchondromas. Current evidence supports a measurable prevalence of concurrent gliomas in patients with Ollier's disease. In patients with Ollier's disease, isocitrate dehydrogenase 1 (IDH1) and isocitrate dehydrogenase 2 (IDH2) mutations were shown to be a predisposing factor to the development of astrocytomas in conjunction with subsequent mutations in adenosine triphosphate (ATP)-dependent helicase ATRX (ATRX) and tumor protein p53 (TP53). We present the case report of a 21-year-old man with an adolescent diagnosis of Ollier's disease who developed a grade II astrocytoma in early adulthood. The patient presented to the emergency room with seizures, was given anti-seizure medication, and later underwent chemotherapy and resection of the astrocytomas. A literature review showed 12 additional patients diagnosed with Ollier's disease who were subsequently diagnosed with a grade II astrocytoma later in life. After reviewing the genetics behind Ollier's disease, the importance of performing routine cranial magnetic resonance imaging (MRI) in these patients becomes apparent.

## Introduction

Ollier's disease (enchondromatosis) is a nonheritable dysplasia of cartilage characterized by multiple enchondromas with a prevalence of 1:100,000 [1,2]. The clinical presentation of Ollier's disease varies, but the primary reported symptoms include bone pain, palpable nodules of the extremities, discrepancy in limb lengths, and bone fractures [3]. Another disease characterized by multiple enchondromas, called Maffucci syndrome, is differentiated by having bilateral enchondroma distribution, vascular overgrowths, and soft tissue hemangiomas [4]. Tumors, such as cholangiocarcinoma, pituitary adenomas, juvenile granulosa tumors, and gliomas, develop in around 50% of cases of Ollier's disease and Maffucci syndrome [5]. Literature suggests that gliomas in particular occur in patients with Ollier's disease, with a prevalence of 5% and a median age of diagnosis of 23.7 years [6,7]. Astrocytomas are the most prevalent type of central nervous system (CNS) tumor associated with Ollier's disease [2]. Clinical and molecular literature analyses emphasize the prevalence of the comorbidity of both Ollier's disease and astrocytomas compared to a non-Ollier's astrocytoma. Clinically, the region of presentation differs, and on a molecular level, mutations in the isocitrate dehydrogenase (IDH), adenosine triphosphate (ATP)-dependent helicase ATRX (ATRX), and tumor protein p53 (TP53) genes help link astrocytomas to Ollier's disease [1,2,8,9].

Here, we present the case of a 21-year-old man with Ollier's disease who developed a grade II astrocytoma. Because neurological tumors in Ollier's disease are often diagnosed only after symptom onset, earlier radiologic surveillance may allow detection at a more treatable stage.

This article was previously presented as a poster at the 2025 Internal Neurology and Brain Disorders Conference Research Consortium on October 22, 2025, and the 2024 Kansas City Medical Conference Annual Meeting on December 7, 2024.

## Case presentation

**Figure 1:**
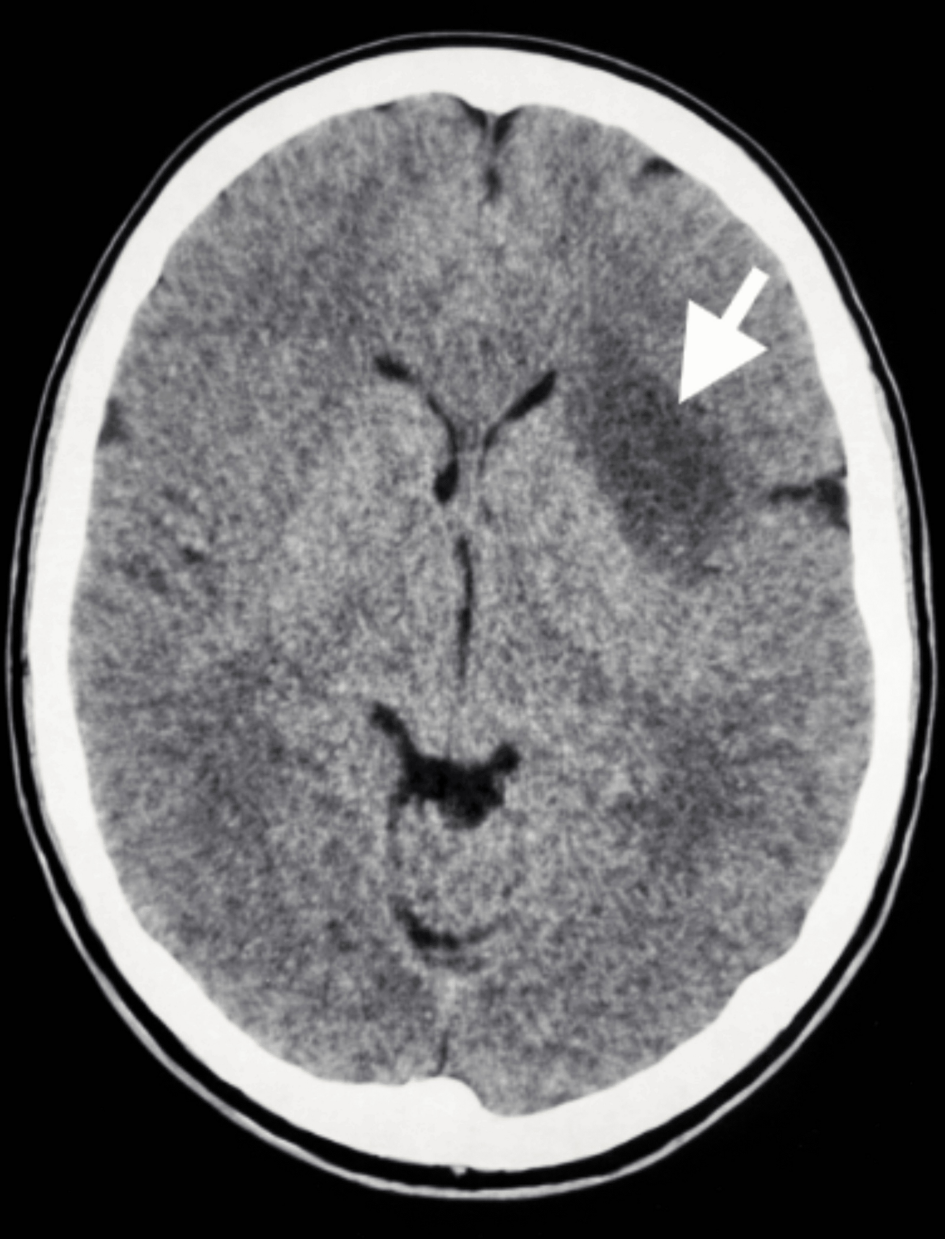
Axial non-contrast CT image of the brain. The white arrow indicates an area of low density seen in the left anterior aspect of the external capsule that extended to the anterior insular cortex and frontal white matter. This is consistent with a low-attenuation lesion suggestive of a low-grade tumor CT: computed tomography

**Figure 2:**
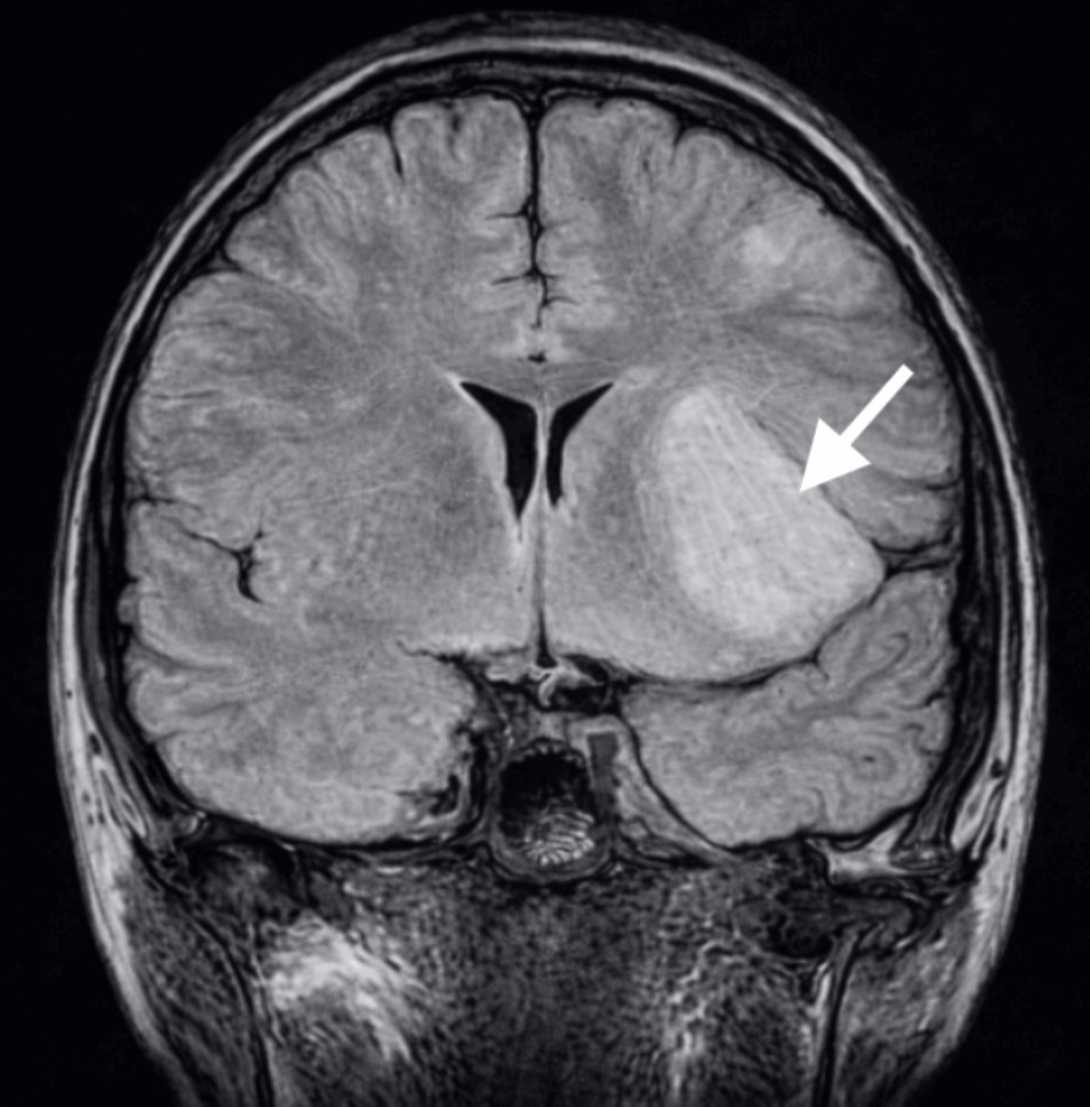
Coronal FLAIR MRI of the brain without contrast demonstrates a poorly circumscribed, FLAIR-hyperintense lesion in the left anterior aspect of the external capsule extending into the anterior insular cortex and frontal white matter (white arrow), imaging features suggestive of a low-grade astrocytoma FLAIR: fluid-attenuated inversion recovery; MRI: magnetic resonance imaging

**Figure 3:**
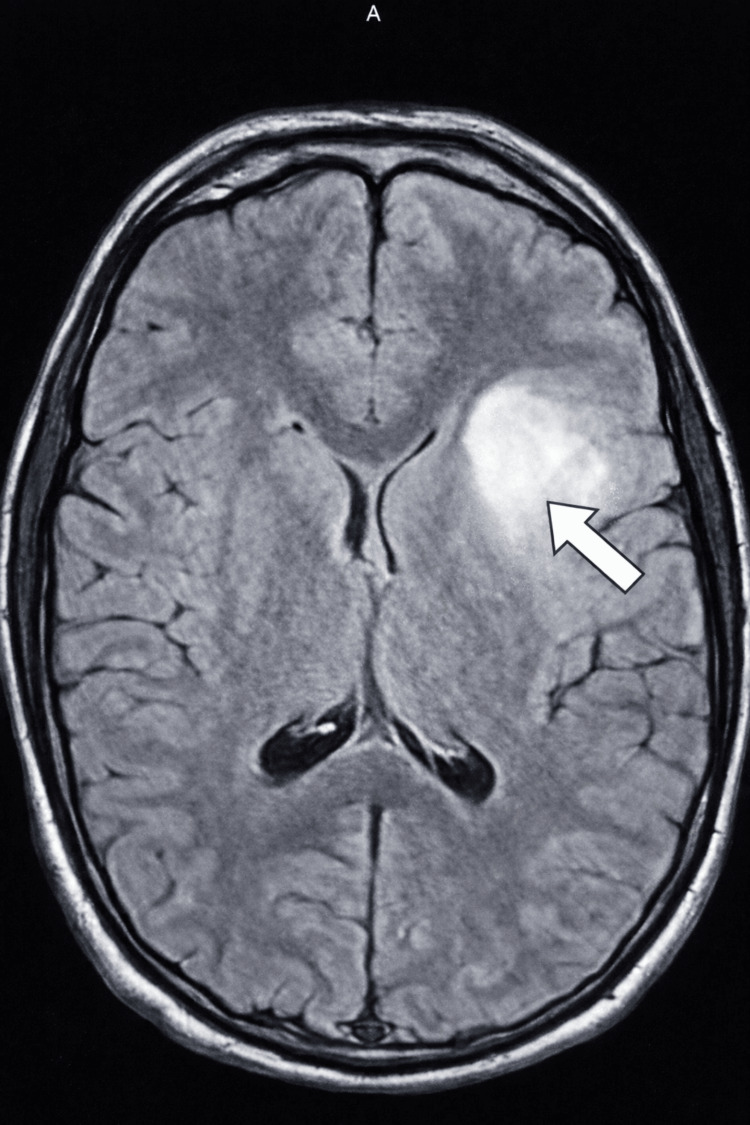
Axial FLAIR MRI of the brain without contrast demonstrates a FLAIR-hyperintense lesion in the left anterior aspect of the external capsule extending into the anterior insular cortex and adjacent frontal white matter (arrow), with imaging features consistent with a low-grade glioma FLAIR: fluid-attenuated inversion recovery; MRI: magnetic resonance imaging

**Figure 4:**
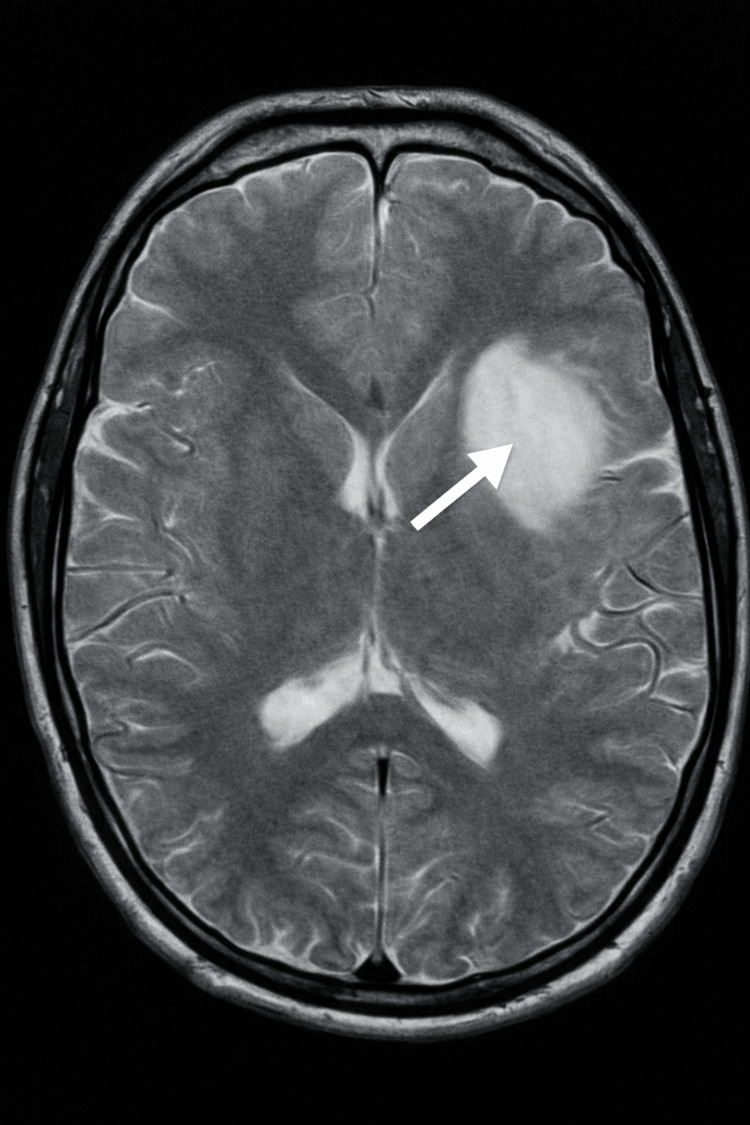
Axial T2-weighted MRI of the brain demonstrates a T2-hyperintense lesion in the left anterior external capsule extending into the anterior insular cortex and frontal white matter (arrow), with imaging features consistent with a low-grade glioma MRI: magnetic resonance imaging

In October 2023, a 21-year-old man presented with seizure-like symptoms to the emergency department. The patient experienced the seizure-like activity for approximately three minutes. Past medical history included Ollier's disease with surgical interventions to remove multiple enchondromas on both of his hands. Vital signs were stable, and the complete blood count was within standard ranges. The complete metabolic panel showed two minor abnormalities, which were clinically insignificant. Neurological evaluation was normal. A computed tomography (CT) scan of the head was ordered, and an area of low density was seen in the left anterior aspect of the external capsule that extended to the anterior insular cortex and frontal white matter (Figure 1). This area measured 4.3 × 3 × 3 cm and was suspected to be an infarct or mass. Magnetic resonance imaging (MRI) scans with and without contrast were subsequently ordered and showed two lesions suspected to be low-grade gliomas (Figures 2-4). The patient was discharged with a prescription of levetiracetam for seizure management and was recommended for an outpatient follow-up biopsy of the masses. Below are the MRI and CT images with and without contrast of the astrocytomas.

In December 2023, two months following the emergency room visit, the patient was referred to an outside university hospital and was diagnosed with multiple grade II astrocytomas of the brain. No IDH, ATRX, TP53, or 1p/19q genetic testing was completed for this patient. The patient underwent chemotherapy and resection of the astrocytomas. He was prescribed ivosidenib to prevent the progression of the astrocytomas and continued taking levetiracetam. The patient returned to the emergency department in February 2024 for the evaluation of a seizure. The seizure occurred for one minute, and the patient was in a postictal state for 30 minutes. The patient stated he had only three or four seizures after his initial diagnosis of astrocytomas in December until the current visit. Laboratory findings and vital signs were within normal limits. Neurological examination was normal. The patient was discharged with lacosamide for seizure management and counseled on breakthrough seizures. The patient passed away in July 2025.

## Discussion

Including our present case, only 33 recorded cases of Ollier's disease caused brain gliomas to our knowledge [1]. Of those 33 recorded cases, we focused our analysis on the 12 patients who specifically presented with grade II astrocytomas [1,6]. Ollier's disease presents with multiple enchondromas and a variety of CNS neoplasms. To our knowledge, there are a total of 12 cases of Ollier's disease presenting with grade II astrocytomas, including the present case. We present these 12 cases in Table 1 and Table 2 below, detailing history, treatment, and prognosis [1,3,4,7,10-16].

**Table 1:** Case presentations of grade II astrocytomas caused by Ollier's disease Data are sourced from previously published studies [1,3,4,7,10-16]. M: male; F: female; IDH1: isocitrate dehydrogenase 1

Patient	Authors/year	Sex/age diagnosed with astrocytoma	Age diagnosed with Ollier's disease	Histology	Molecular analysis
1	Mellon et al., 1988 [10]	M/34	3	Astrocytoma (grade II)	Not specified
2	Patt et al., 1990 [11]	M/24	5	Astrocytoma (grade II)	Not specified
3	Hofman et al., 1998 [12]	M/28	8 months	Astrocytoma (grade II)	Not specified
4	van Nielen and de Jong, 1999 [4]	M/28	Early in childhood	Astrocytoma (grade II)	Not specified
5	Şimşek et al., 2002 [16]	F/24	7	Astrocytoma (grade II)	Not specified
6	Mahafza, 2004 [13]	F/21	9	Astrocytoma (grade II)	Not specified
7	Bathla et al., 2012 [7]	M/16	15	Astrocytoma (grade II)	IDH1 mutation positive
8	Pearce et al., 2012 [14]	M/19	Previously diagnosed, not specified	Low-grade astrocytoma	IDH1 mutation negative
9	Khan et al., 2013 [3]	M/35	9	Low-grade astrocytoma	Not specified
10	Rumeh et al., 2020 [15]	F/23	Not specified	Diffuse astrocytoma (grade II)	IDH1 mutation positive
11	Corvino et al., 2022 [1]	M/33	2	Diffuse astrocytoma (grade II)	IDH1 mutation positive
12	Present case	M/21	Previously diagnosed, not specified	Astrocytoma (grade II)	Not specified

**Table 2:** Patient details of astrocytoma site, neurological presentation, intervention, and outcome Data are sourced from previously published studies [1,3,4,7,10-16]. CN: cranial nerve

Patient	Authors	Intracranial location	Neurological signs and symptoms	Intervention	Outcome
1	Mellon et al., 1988 [10]	Right frontal lobe	Presenting symptoms: 3-year history of seizures. Neurological exam: normal	Craniotomy; resection	Not specified
2	Patt et al., 1990 [11]	Brainstem	Presenting symptoms: nausea; neurologic onset at age 21. Cranial nerve deficits: CN VI palsy; CN VII-XI right-sided deficits. Motor: slight right hemiparesis. Cerebellar: hemiataxia	Biopsy	Not specified
3	Hofman et al., 1998 [12]	Multicentric (left temporal lobe, brainstem)	Presenting symptoms: 2-year history of diplopia and headaches; worsening over 6 months. Cranial nerve deficits: left CN VI palsy. Cerebellar: coordination disturbance. Motor/reflex: right Babinski sign	Biopsy; radiotherapy	Alive at 1 year
4	Van Nielen and de Jong, 1999 [4]	Multicentric (left temporal lobe, brainstem)	Presenting symptoms: diplopia on left gaze; choking; imbalance. Cranial nerve deficits: CN VI palsy; subtle CN XI and XII deficits. Motor/reflex: pyramidal signs; brisk reflexes; ankle clonus; Babinski	Biopsy; radiotherapy	Not specified
5	Şimşek et al., 2002 [16]	Right frontal lobe	Presenting symptoms: seizures; headache. Neurological exam: normal	Resection	Alive at 3 years post-operative
6	Mahafza, 2004 [13]	Multicentric (right frontal lobe, brainstem)	Presenting symptoms: 2-month diplopia (left eye); 5-month progressive right-sided weakness. Cranial nerve deficits: left CN VI palsy. Motor: bilateral limb weakness (3/5); brisk reflexes; right Babinski	Biopsy	Not specified
7	Bathla et al., 2012 [7]	Multicentric (frontal lobe, right insular cortex, right cingulate gyrus)	Presenting symptoms: 2-week intermittent headache	Biopsy; craniotomy at progression; radiotherapy	Deceased at 8 years
8	Pearce et al., 2012 [14]	Multicentric (frontal lobe, parieto-occipital junction)	Presenting symptoms: 3-week persistent headache; later headache and vomiting.Cranial nerve/sensory deficits: right hearing deficit; decreased visual acuity; decreased sensory acuity; hand paresthesias	Biopsy; debulking; radiotherapy; frontal lobectomy after progression	Not specified
9	Khan et al., 2013 [3]	Left insular cortex	Presenting symptoms: 1-year intermittent headache and giddiness; loss of consciousness. Motor: involuntary hand movements. Autonomic: loss of bowel and bladder control	Biopsy; surgery	Not specified
10	Rumeh et al., 2020 [15]	Multicentric (left frontal lobe)	Presenting symptoms: 2-month frontal pressure headache; two 15-minute seizures	Craniotomy	Not specified
11	Corvino et al., 2022 [1]	Multicentric (frontal lobe, temporal lobe, insular cortex)	Presenting symptoms: epileptic seizure; partial motor seizures after progression	Biopsy; radiotherapy; temozolomide; chemotherapy	Alive at 5 years
12	Present case	Multicentric (frontal lobe, insular cortex)	Presenting symptoms: seizure leading to astrocytoma diagnosis. Neurological exam: normal	Microsurgical resection; ivosidenib; chemotherapy; resection	Deceased at 1.5 years

The patients outlined in Table 1 and Table 2 were often diagnosed with Ollier's disease in early childhood and diagnosed with astrocytomas later in life. Of the eight patients with a specified date of diagnosis in Table 1, there was a 20.54-year difference between the initial diagnosis of Ollier's disease and the detection of astrocytoma. The frontal lobe, brainstem, and insular cortex were the most common sites for astrocytoma involvement, many of which were multicentric. The most common signs and symptoms these patients presented with were seizures (5/12), headaches (6/12), motor weakness (2/12), Babinski reflex (3/12), disturbance in coordination (3/12), cranial nerve palsies (4/12), and notably diplopia (double vision) (3/12). Of these, sixth nerve palsy (4/12) is the most frequent neuro-ophthalmic finding reported in patients with Ollier's disease [17]. These symptoms, along with proptosis, hoarseness, dysarthria, and facial dysaesthesia, are the most common clinical signs in the current literature [17].

In patients with Ollier's disease who developed a grade II astrocytoma, the average survival following diagnosis was 3.7 years, based on available data from five of the 12 cases included in Table 2. Survival information was unavailable for the remaining patients due to loss to follow-up. In the broader literature, the overall survival for grade II astrocytoma has been reported to range from seven to eight years [18].

Additionally, astrocytoma location appears to differ in patients with Ollier's disease. In this population, astrocytomas are typically diffuse, low-grade, and most commonly located in the frontal lobe and brainstem, consistent with our tabulated findings. In contrast, astrocytomas in patients without Ollier's disease more frequently involve the temporal lobe and demonstrate a wider range of tumor grades [1,7].

The prognosis of Ollier's disease with brain gliomas is not well defined, and presenting with gliomas is a rarity in itself. Many patients are lost to follow-up deeming their outcome unknown. A retrospective review of patients with Ollier's disease and brain gliomas found that of 31 patients, 15 were alive, three were deceased, and the rest of the patients had an unknown outcome [1]. To add to the literature, our patient with a grade II astrocytoma underwent chemotherapy and is now deceased. Based on our cohort of 12 patients with grade II astrocytomas and concurrent Ollier's disease, along with 31 previously reported cases of Ollier's disease-associated brain gliomas, there is not enough data to support a clear mortality rate, quality of life, or prognosis in patients with brain gliomas associated with Ollier's disease.

Genetic research reports that mutations in the isocitrate dehydrogenase 1 (IDH1) and the isocitrate dehydrogenase 2 (IDH2) genes are prevalent in both enchondromas and gliomas. Patients with Ollier's disease are more prone to malignancies in other regions of the body due to somatic IDH mosaicism [8]. Of those with Ollier’s, around 81% were shown to have mutations in the IDH1 or IDH2 genes [8]. The IDH1 mutation is also seen in up to 71% of grade II gliomas [19]. In the present case, genetic testing was not performed. Among the cases summarized in Table 2, genetic testing results were reported for four patients, with three testing positive for an IDH1 mutation. Molecular data were not available for the remaining cases, primarily because their presentations were diagnosed before routine molecular profiling. In two additional cases, including the present case, diagnoses occurred during the molecular era, but genetic testing was not performed. Collectively, these findings support a potential shared molecular pathway involving IDH mutations that may be relevant to the development of astrocytoma in patients with Ollier's disease.

In this case, Ollier's disease coincided with the development of a grade II astrocytoma, a slow-growing brain neoplasm of the glial cells. More specifically, a grade II astrocytoma is defined as a diffuse glioma with an IDH1 or IDH2 mutation that most likely has TP53 and ATRX mutations, without a 1p/19q codeletion [9]. These molecular features typically present as a strong TP53 immunoreactivity and a loss of ATRX expression [20]. TP53 mutations contribute to the dominant-negative inhibition of wild-type TP53, a nuclear transcription factor with pro-apoptotic function [21]. While TP53 mutations are common, having over a 50% prevalence in human cancers, they seem to be especially relevant in patients with Ollier's disease and gliomas. In a 2020 study, five out of six patients who had Ollier's disease and gliomas had TP53 expression [21]. ATRX mutations, on the other hand, contribute to abnormal maintenance in telomeres, leading to the alternative lengthening of telomeres [20]. While chromatin-remodeling pathways, like ATRX, are common in gliomas, with 80% of them being detected in secondary gliomas, ATRX mutations tend to be specific to astrocytic tumors carrying IDH1 and IDH2 mutations [2].

These ATRX and TP53 mutations may play a key role in explaining the development of gliomas, and specifically astrocytomas, in patients with Ollier's disease. IDH mutations, being one of the earliest oncogenic events in a majority of low-grade gliomas, are sufficient to induce enchondromas in animal trials [20]. However, IDH mutations alone have not been sufficient to induce gliomas and require additional alterations, like ATRX and TP53 [20]. This could elucidate why enchondromas appear before gliomas in patients with Ollier's and justify the importance of regular cerebral MRI scans in the follow-up appointments of patients with Ollier's disease [20].

## Conclusions

We present a 21-year-old man with Ollier's disease who developed a grade II astrocytoma involving the insular cortex and frontal lobe, with multiple neoplasms treated by resection and chemotherapy. IDH mutations, often the earliest oncogenic event in low-grade gliomas, are frequently observed with grade II gliomas, while ATRX and TP53 mutations are commonly associated with astrocytoma development. Physicians should remain attentive to the early signs of grade II astrocytoma in patients with Ollier's disease, including seizures, headaches, motor or sensory deficits, and cranial nerve involvement affecting vision or hearing. Because astrocytomas in Ollier's disease are frequently diagnosed only after significant symptoms arise, consideration of routine cranial MRI surveillance following diagnosis may allow earlier detection and improved outcomes, though further studies are needed to define optimal surveillance strategies and clinical impact.

## References

[1] Corvino S, Mariniello G, Corazzelli G (2022). Brain gliomas and Ollier disease: molecular findings as predictive risk factors?. Cancers (Basel).

[2] Gajavelli S, Nakhla J, Nasser R, Yassari R, Weidenheim KM, Graber J (2016). Ollier disease with anaplastic astrocytoma: a review of the literature and a unique case. Surg Neurol Int.

[3] Khan SH, Rather TA, Koul PA, Makhdoomi R, Bhat AR, Malik D, Manohar R (2013). Bone scintigraphy in Ollier's disease: a rare case report. Indian J Nucl Med.

[4] van Nielen KM, de Jong BM (1999). A case of Ollier's disease associated with two intracerebral low-grade gliomas. Clin Neurol Neurosurg.

[5] El Abiad JM, Robbins SM, Cohen B, Levin AS, Valle DL, Morris CD, de Macena Sobreira NL (2020). Natural history of Ollier disease and Maffucci syndrome: patient survey and review of clinical literature. Am J Med Genet A.

[6] Hori K, Matsumine A, Niimi R, Maeda M, Uchida K, Nakamura T, Sudo A (2010). Diffuse gliomas in an adolescent with multiple enchondromatosis (Ollier's disease). Oncol Lett.

[7] Bathla G, Gupta S, Ong CK (2012). Multifocal intracranial astrocytoma in a pediatric patient with Ollier disease. Indian J Radiol Imaging.

[8] Karabulut AK, Türk S, Tamsel İ, Kim J, Argın M (2021). Diffuse midline glioma in Ollier disease: a case report and a brief review of the literature. Radiol Case Rep.

[9] Otani R, Uzuka T, Higuchi F (2018). IDH-mutated astrocytomas with 19q-loss constitute a subgroup that confers better prognosis. Cancer Sci.

[10] Mellon CD, Carter JE, Owen DB (1988). Ollier's disease and Maffucci's syndrome: distinct entities or a continuum. Case report: enchondromatosis complicated by an intracranial glioma. J Neurol.

[11] Patt S, Weigel K, Mayer HM (1990). A case of dyschondroplasia associated with brain stem glioma: diagnosis by stereotactic biopsy. Neurosurgery.

[12] Hofman S, Heeg M, Klein JP, Krikke AP (1998). Simultaneous occurrence of a supra- and an infratentorial glioma in a patient with Ollier's disease: more evidence for non-mesodermal tumor predisposition in multiple enchondromatosis. Skeletal Radiol.

[13] Mahafza WS (2004). Multiple enchondromatosis Ollier's disease with two primary brain tumors. Saudi Med J.

[14] Pearce P, Robertson T, Ortiz-Gomez JD, Rajah T, Tollesson G (2012). Multifocal supratentorial diffuse glioma in a young patient with Ollier disease. J Clin Neurosci.

[15] Rumeh AS, Shaikh T, Lari A, Muharib AA, Al Shakweer W (2020). Diffuse astrocytoma and Ollier's disease. J Neurol Res.

[16] Şimşek S, Seçkin H, Belen D, Erekul S (2002). Intracranial glioma. Türk Nöroşirürji Dergisi.

[17] Raoof N, Batty R, Carroll TA, Pepper IM, Sandison A, Eckersley R, Hickman SJ (2015). Relapsing-remitting sixth nerve palsy in association with Ollier's disease. Neuroophthalmology.

[18] Pansuriya TC, van Eijk R, d'Adamo P (2026). Somatic mosaic IDH1 and IDH2 mutations are associated with enchondroma and spindle cell hemangioma in Ollier disease and Maffucci syndrome. Nat Genet.

[19] Labussiere M, Sanson M, Idbaih A, Delattre JY (2010). IDH1 gene mutations: a new paradigm in glioma prognosis and therapy?. Oncologist.

[20] Antonelli M, Poliani PL (2022). Adult type diffuse gliomas in the new 2021 WHO Classification. Pathologica.

[21] Nishtha Y, Maya B, Shetty SS, Ganaraj VH, Nupur P, Yasha TC, Netravathi M (2020). A case of Ollier's disease with P53 mutation positive and IDH1 (R132H) negative multicentric gliomas. Neurol India.

